# Generation of multi-scrolls in corona virus disease 2019 (COVID-19) chaotic system and its impact on the zero-covid policy

**DOI:** 10.1038/s41598-023-40651-2

**Published:** 2023-08-25

**Authors:** Muhammad Marwan, Maoan Han, Rizwan Khan

**Affiliations:** 1 School of Mathematical Sciences, Zhejiang Normal University, Jinhua, 321004 China; 2 School of Computer Science, Zhejiang Normal University, Jinhua, 321004 China

**Keywords:** Control theory, Differential equations, Biomedical engineering, Applied mathematics

## Abstract

In this paper, we discussed the impossibility of achieving zero-covid cases per day for all time with the help of fuzzy theory, while how a single case can trigger chaotic situation in the nearby city is elaborated using multi-scrolls. To accomplish this goal, we consider the number of new cases per day; $x_{1}$ to be the preferred state variable by restricting its value to the interval (0, 1). One can need to think of $x_{1}$ as a member of a fuzzy set and provide that set with appropriate membership functions. Moreover, how a single incident in one city can spread chaos to other cities is also addressed at length, using multi-scroll attractors and the signal excitation function. In addition, a bifurcation diagram of daily new instances vs the parameter $\alpha _{1}$ is shown, elaborating that daily new cases may show a decrease under strict rules and regulations, but can again lead to chaos. Apart from biologist, this paper can play vital role for engineers as well in a sense that, a signal function can be embedded in non-symmetric systems for the creation of multi-scroll attractors in all directions using a generalized algorithm that has been designed in the current work. Finally, it is our future target to show that the covid is leading towards influenza and will be no more dangerous as was in the past.

## Introduction

The degree of chaos and unpredictability in a system is determined by its sensitivity to the initial conditions, the bifurcation parameter, and the dense oscillatory solutions. The aforementioned features of chaos in continuous systems are increasing its appeal by using it as a transmitter in secure communication^1^, path planing problems^2–4^, cryptography^5^ and motion control^6^ but apart from these applications in engineering, the term ”chaos” continues to be associated with negative impact in biological models and still remain as a villain such as Cancer^7^, Ebola^8,9^, Influenza^10,11^, HIV^12,13^ and Parkinson^14^ epidemic diseases models.

The birth of corona virus disease (covid) in Wuhan has altered the global level of living and since its emergence, researchers have worked on it from various perspectives. In the early 2020, an SEIR-based model of COVID-19^15^ including some means of control was developed by He et al. Then, based on the location data, Yan and Lan^16^ built a model where people were more likely to engage. Batabyal^17^ in 2020 discussed the impact of quarantine and lock-down on the deadly virus in a connection with stability analysis. Mathematically, pandemic and epidemic diseases are designed on the bases of Susceptible, Infectious & Recovered (SIR) or Susceptible, Exposed, Infectious & Recovered (SEIR) based models and similar COVID-19 models can be studied in the book of Tanimoto^18^, where he elaborated the design of this pandemic disease in depth using SIR and SEIR based models. Machado and Ma^19^ worked on the forecast of COVID-19 and examined its nonlinear dynamics. Meanwhile, the transmission rate of this disease in Henan province was measured by Li *et al*^20^. In 2020, Li et al^21^ discussed the after shocks of pandemic disease among teaching community which not even disturbed the education sector but has also produced a lot of anxiety among them, whereas in 2022 Hu et al^22^ briefly explained the impact of COVID-19 on the digital trading in China. In similar fashion, Xie et al^23^ designed a new statistical based model for COVID-19 model and analyzed it for small and medium-sized enterprises using Bayesian network. There are a lot of models designed for COVID-19 but the most recent chaotic can be found in the work of Mangiarotti *et al*^24^.

Researchers are continuing to investigate covid from variety of angles, proving its continued importance as a topic of study. Many researchers have recently shown considerable interest in a fractional−order version of the covid model^25–28^. The most recent work on covid-19 can be found in the work of^29^, where they have determined spread of infection rate using superposition rule of Gaussian pulses and informed the world by elaborating the occurrence of multiple waves. The speed rate of this disease was high, that is why, Hammad et al^30^ implemented a technique, based on image processing, for the fast detection of covid disease and its controlling. In 2021, Paul et al^31^ worked on the fast emergence of covid by introducing two parameters-based model, while Photiou^32^ gathered data from the social media and informed the world about the spread of this pandemic disease. Till-now, many lives have been lost to this disease, and alarming rates of mortality have been reported in Italy, the United States, and South Korea. However, China has introduced a new term—”smart lock-down”—that has had a profound effect on containing the virus. In order to combat covid, the majority of countries have adopted the same strategy. During this time, a number of vaccines were also developed and implemented; last year in December, 68.4% of the global population has received at least one vaccination^33^. The next goal of China was the reduction of covid to zero per day across the country, after having achieved great success in the implementation of smart lock-down. Since more than half the world’s population has been immunized, the incidence of covid is low, and fatalities have decreased. There is still a problem, though, with the influx of new cases. Many precautions are taken, and special applications for smartphones were developed with the goal of reaching a state of “zero-covid”, but even so, a single instance of the disease can suddenly appear and have a profound impact on a huge number of people all at once.

The reduction of new cases of covid is a popular goal of suppression efforts. Because of its ability to deal with stability and parameter tuning in conjunction with the expression of nonlinear terms in any system in a linearized way, the concept of Takagi–Sugeno (**TS**) fuzzy systems is necessary for this purpose. This article explains how to transform chaotic (Lorenz, Flexible joint robot arm, Duffing oscillator, and Rossler) systems into deterministic (**TS**) ones (Chapter 6,^34^). In addition to their usefulness, fuzzy models can be applied in many other contexts as well. The fuzzy based drive and slave chaotic systems are synchronized with the aid of finite time command filter in 2021 by Alassafi et al.^35^. In 2022, Babanli et al.^36^ modeled fuzzy chaotic system and implemented it as an application in secure communication. A fuzzy wind turbine systems^37^ are controlled for networked systems using H$_{\infty }$ controller.

When a signal function is added to a chaotic system, many such attractors can appear in two or three dimensions, a phenomenon known as a multi-scroll attractor. Elvakil et al.^38^ took into account a double wing attractor and found a case of mirror plane symmetric chaotic attractor in a modified Lorenz system. New techniques for producing multi-scroll attractors have been developed since this work was published^39–41^. For the past decade, scientists have experimented with various signal functions, such as switching control^42^, piece-wise hysteresis function^43^, shifting transformation^44^, saw−tooth function^45,46^ and train pulses^47^, to generate multiple scrolls. The introduction of Multilevel-logic pulse sources by Hong *et al*^48^ into chaotic systems directed researchers in a new way of thinking about the generation of multi-scrolls. More generalization was brought to the excitation of multilevel-logic pulse attractors in the satellite system by Anam *et al*^49^. Before explaining why we’re doing this, it’s worth noting that all previous work on multi-scrolls in non-symmetric systems has focused on chaotic systems that obey the property of symmetry in the state variables.

From the above discussion about COVID-19 and cited work we observed that it is an important topic, but after studying literature about multi-scrolls we noticed that:

- A chaotic system with at least two attractors achieves multi-scrolls all the time, but what about those systems bearing a single attractor?
- Is it possible for a country or region to achieve zero covid cases for all the time?

Keeping in mind these issues motivated us to implement a suitable signal function into chaotic systems based on a single wing. That is why, we have selected a single winged chaotic system reflecting one of the main issues in the current time. The second question is also an integral part of this work where we have used the concepts of multi-scrolls and fuzzy sets in combination to elaborate that the zero covid cases throughout the life is impossible. This scenario is deeply discussed with the aid of fuzzy theory, where the number of new cases per day is restricted to the interval (0, 1) and its spreading speed is visualized using three dimensional multi-scrolls.

## Results

### Covid-19 model

There are many models introduced for pandemic disease, but in 2021, a covid model^24^ was introduced by considering data taken from highly infected countries such as Italy, Japan, South Korea and China has got much importance. The considered model taken from^11,24^ is given as :

1 $$\begin{aligned} \dot{x}_{1}&=\alpha _{1} x_{3}-\alpha _{2} x_{3}^2+\alpha _{3} x_{3}x_{2} -\alpha _{4} x_{1}+\alpha _{5} x_{1} x_{3}-\alpha _{6} x_{1} x_{2}, \nonumber \\ \dot{x}_{2}&=\beta _{1} x_{2} x_{3}-\beta _{2} x_{1} x_{2}, \nonumber \\ \dot{x}_{3}&=\gamma _{1} x_{3}-\gamma _{2} x_{1} x_{3}-\gamma _{3} x_{1} x_{2}+\gamma _{4} x_{1}^2, \end{aligned}$$

where $x_{1}$, $x_{2}$, $x_{3}$ shows the daily numbers of new cases, daily additional severe cases and new death cases, respectively. Mathematically, system (1) exhibits chaotic behavior for initial conditions (184, 30, 8) and parameter values given in Table 1. The most affected countries with respect to the number of new cases per day are listed in Table 2 since the exposure of covid-19 to the world. Several countries have implemented various restriction rules to overcome the spread of this disease in their own way. The most effective technique was adopted by China in which they have introduced smart lock-down in a circle of some suitable radius. As the residence in China is based on districts, which are further divided into large number of small communities. Therefore, the technique of smart lock-down was very effective and become very popular such that other countries also adopted their policy to control rapid spreading of corona virus.

**Table 1:** Parametric values for covid-19 system (1).

Parameters	Values	Parameters	Values Values
$\alpha _{1}$	66	$\beta _{1}$	0.05507
$\alpha _{2}$	1.6966	$\beta _{2}$	0.0008238
$\alpha _{3}$	0.148	$\gamma _{1}$	0.31303
$\alpha _{4}$	0.8763	$\gamma _{2}$	0.0001057
$\alpha _{5}$	0.022843	$\gamma _{3}$	$1.008\times 10^{-5}$
$\alpha _{6}$	0.0017342	$\gamma _{4}$	$1.734\times 10^{-6}$

**Table 2:** List of the affected countries with respect to the number of new cases in December, 2022.

Countries	Total cases	New cases	Total deaths	New deaths	Severe cases
South Korea	26,654,729	+ 72,873	30,111	+45	461
Taiwan	8,193,072	+ 18,179	14,029	+38	–
Australia	10,564,087	+ 3807	16,007	+27	68
China	290,787	+ 2225	5,231	+2	110
Kazakhstan	1,395,857	+ 56	13,693	–	24
Pakistan	1,574,939	+ 25	30,630	–	46
Laos	216,492	+ 24	758	–	–
Cambodia	138,035	+ 3	3056	–	–
USA	100,251,354	–	1,102,915	–	2749
India	44,670,787	–	530,591	–	698

*Zero-Covid policy* After trying out lock-down with positive results, of course. The Chinese government has set its sights on the new milestone of zero-covid and made significant progress toward taming this pandemic disease. Following this, we shall demonstrate that a policy of zero-covid is not only unachievable, but also remains dangerously close to chaos.

### Zero-covid policy still exhibit chaos

In this section, we have tried to prove that if the new cases of covid approaches to zero then, there are still chances of chaos. To achieve this possibility, we use the concept of fuzzy theory to transform our considered system into fuzzy covid model and restrict the number of new cases to the interval; **Z**=(0,1).

#### Theorem 1

*Suppose the number of new cases per days in system* (1) *is re-scaled to the interval* **Z*** = (0, 1) using membership functions:*

2 $$\begin{aligned} {\left\{ \begin{array}{ll} \Gamma _{1}=\frac{-x_{1}+M_{2}}{M_{2}-M_{1}}\\ \Gamma _{2}=\frac{x_{1}-M_{1}}{M_{2}-M_{1}} \end{array}\right. } \end{aligned}$$

satisfying the inequality $M_{1}<x_{1}<M_{2}$. Then, the fuzzy dynamical systems (7) and (8) are chaotic as well.

#### *Proof*

To proof Theorem (1), we need to convert system (1) into the following matrix form:

3 $$\begin{aligned} \dot{{\textbf {X}}}=\mathscr {A}{} {\textbf {X}}+\mathscr {B}\mathscr {F}({\textbf {X}}), \end{aligned}$$

where $${\textbf {X}}=(x_{1},x_{2},x_{3})^{T}$$

4 $$\begin{aligned}{}&\mathscr {A}=\begin{pmatrix} -\alpha _{4} &{} 0 &{} \alpha _{1}\\ 0 &{} 0 &{} 0\\ 0 &{} 0 &{} \gamma _{1} \end{pmatrix},\hspace{5.0pt}\mathscr {B}=\begin{pmatrix} 1 &{} 0 &{} 0\\ 0 &{} 1 &{} 0\\ 0 &{} 0 &{} 1 \end{pmatrix}\hspace{5.0pt}\text {and}\hspace{5.0pt}\mathscr {F}({\textbf {X}})=\begin{pmatrix} -\alpha _{2}x_{3}^2 + \alpha _{3}x_{2}x_{3}+\alpha _{5}x_{1}x_{3}- \alpha _{6}x_{1}x_{2}\\ \beta _{1} x_{2} x_{3}-\beta _{2} x_{1} x_{2} \\ -\gamma _{2} x_{1} x_{3}-\gamma _{3} x_{1} x_{2}+\gamma _{4} x_{1}^2 \end{pmatrix}. \end{aligned}$$

Next, we suppose that $x_{1}$; the number of new cases of covid belongs to the set $\textbf{Z}=(0,1)$. Then, the nonlinearities $x_{1}x_{2}$ and $x_{1}x_{3}$ in system (4) needs to be changed as well. Therefore, using Definition 2 and Lemma (1), we get:

5 $$\begin{aligned} {\left\{ \begin{array}{ll} x_{1}x_{2}=(\mu _{1} h_{1} + \mu _{2} h_{2})x_{2}\\ x_{1}x_{3}=(\mu _{1} h_{1} + \mu _{2} h_{2})x_{3}, \end{array}\right. } \end{aligned}$$

where $\mu _{1}=\Gamma _{1}$, $\mu _{2}=\Gamma _{2}$, $h_{1}=M_{1}$ and $h_{2}=M_{2}$. Moreover, we get the membership functions $\Gamma _{1}$ and $\Gamma _{2}$ using Definition 1:

6 $$\begin{aligned} {\left\{ \begin{array}{ll} \Gamma _{1}= \frac{M_{2}-x_{1}}{M_{2}-M_{1}}\\ \Gamma _{2}= \frac{x_{1}-M_{1}}{M_{2}-M_{1}}. \end{array}\right. } \end{aligned}$$

Putting Eq. (5) into Eq. (4) to get the fuzzy version of our considered system. Here, the membership values belong to ${\textbf {Z}}=(0,1)$. Therefore, we define two rules fuzzy-based model of system (1).

**Rule 1:** **IF** $x_{1}$ is about $M_{1}$ **THEN**,

7 $$\begin{aligned} \begin{pmatrix} \dot{x}_{1} \\ \dot{x}_{2} \\ \dot{x}_{3} \end{pmatrix}= \begin{pmatrix} -\alpha _{4} &{} \alpha _{6} M_{1} &{} \alpha _{1}+\alpha _{5} M_{1}\\ 0 &{} -\beta _{2} M_{1} &{} 0\\ 0 &{} -\gamma _{2}M_{1} &{} \gamma _{1}-\gamma _{3}M_{1} \end{pmatrix} \begin{pmatrix} {x}_{1} \\ {x}_{2} \\ {x}_{3} \end{pmatrix} + \begin{pmatrix} -\alpha _{2}x_{3}^2 + \alpha _{3}x_{2}x_{3}\\ \beta _{1} x_{2} x_{3} \\ \gamma _{4} x_{1}^2 \end{pmatrix}. \end{aligned}$$

**Rule 2:** **IF** $x_{1}$ is about $M_{2}$ **THEN**,

8 $$\begin{aligned} \begin{pmatrix} \dot{x}_{1} \\ \dot{x}_{2} \\ \dot{x}_{3} \end{pmatrix}= \begin{pmatrix} -\alpha _{4} &{} \alpha _{6} M_{2} &{} \alpha _{1}+\alpha _{5} M_{2}\\ 0 &{} -\beta _{2} M_{2} &{} 0\\ 0 &{} -\gamma _{2}M_{2} &{} \gamma _{2}-\gamma _{3}M_{2} \end{pmatrix} \begin{pmatrix} {x}_{1} \\ {x}_{2} \\ {x}_{3} \end{pmatrix} + \begin{pmatrix} -\alpha _{2}x_{3}^2 + \alpha _{3}x_{2}x_{3}\\ \beta _{1} x_{2} x_{3} \\ \gamma _{4} x_{1}^2 \end{pmatrix}. \end{aligned}$$

Systems (7) and (8) are the lower and upper fuzzy chaotic versions of our considered model (1).

**Figure 1:**
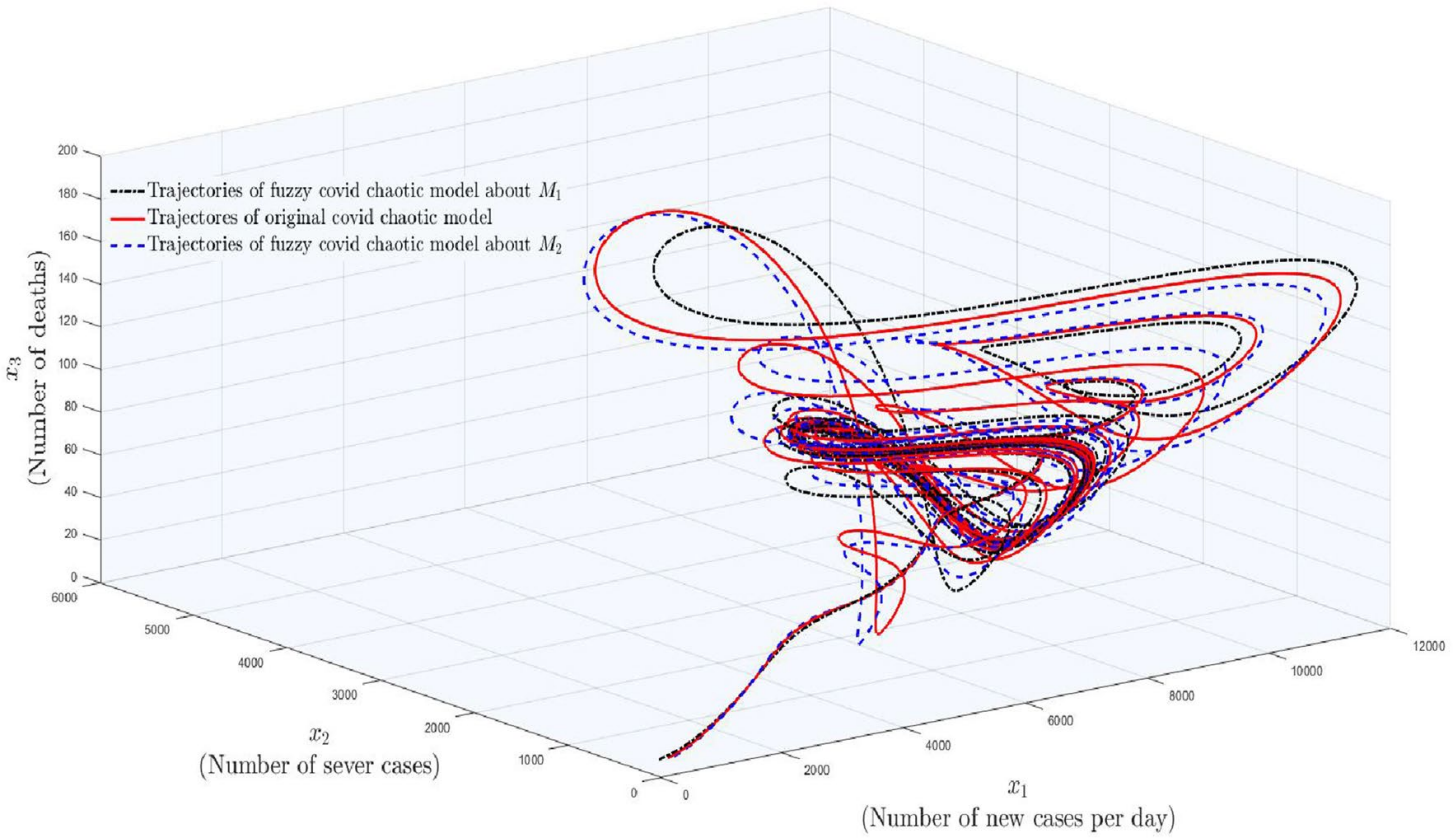
The occurrence of chaos in fuzzy based covid−19 system.

In Fig. 1, one can observe three colored trajectories in which red colored trajectory shows the phase portrait of original COVID-19 model (1), whereas black and dark blue colored dotted curves show the trajectories of fuzzy models obtained in **Rule 1** and **Rule 2**, respectively. From there, one can observe that the fuzzy systems obtained in both rules are still chaotic. Hence, it is proved that, if the number of new cases per day are reduced to the interval (0, 1) then there are still chances of the existence of chaos. $\square$

In those days, the best real-life example of Theorem 0.1 was in China. Because, they were trying to achieve the zero corona cases in the whole country and for this their government had made a lot of great achievements and arrangements. But, only a single case triggered the situation to worst each time not only in the existing city but in the nearby cities as well. Latter on, mathematically, such conditions are deeply explained with the aid of signal pulse function and bifurcation diagram.

#### Theorem 2

*Suppose the number of severe cases per days in system* (1) *is re-scaled to the interval* **Z*** = (0, 1) using membership functions:*

9 $$\begin{aligned} {\left\{ \begin{array}{ll} \Gamma _{1}=\frac{-x_{2}+K_{2}}{K_{2}-K_{1}}\\ \Gamma _{2}=\frac{x_{2}-K_{1}}{K_{2}-K_{1}} \end{array}\right. } \end{aligned}$$

satisfying the inequality $K_{1}<x_{2}<K_{2}$. Then, system (10)

10 $$\begin{aligned} \dot{x}_{1}&=\alpha _{1} x_{3}-\alpha _{2} x_{3}^2+\alpha _{3} x_{3}K_{i} -\alpha _{4} x_{1}+\alpha _{5} x_{1} x_{3}-\alpha _{6} x_{1} K_{i}, \nonumber \\ \dot{x}_{2}&=(\beta _{1} x_{3}-\beta _{2} x_{1}) K_{i}, \nonumber \\ \dot{x}_{3}&=\gamma _{1} x_{3}-\gamma _{2} x_{1} x_{3}-\gamma _{3} x_{1} K_{i}+\gamma _{4} x_{1}^2, \end{aligned}$$

for $i=1,2$ are chaotic as well.

#### Theorem 3

*Suppose the number of deaths per day in system* (1) *is re-scaled to the interval* **Z** *= (0, 1) using membership functions:*

11 $$\begin{aligned} {\left\{ \begin{array}{ll} \Gamma _{1}=\frac{-x_{3}+N_{2}}{N_{2}-N_{1}}\\ \Gamma _{2}=\frac{x_{3}-N_{1}}{N_{2}-N_{1}} \end{array}\right. } \end{aligned}$$

satisfying the inequality $N_{1}<x_{3}<N_{2}$. Then, system (12)

12 $$\begin{aligned} \dot{x}_{1}&=\alpha _{1} x_{3}-\alpha _{2} x_{3}^2+\alpha _{3} N_{i}x_{2} -\alpha _{4} x_{1}+\alpha _{5} x_{1} N_{i}-\alpha _{6} x_{1} x_{2}, \nonumber \\ \dot{x}_{2}&=\beta _{1} N_{i} x_{2}-\beta _{2} x_{1} x_{2}, \nonumber \\ \dot{x}_{3}&=\gamma _{1} x_{3}-\gamma _{2} x_{1} N_{i}-\gamma _{3} x_{1} x_{2}+\gamma _{4} x_{1}^2, \end{aligned}$$

for $i=1,2$ are chaotic as well.

#### Remark 1

Theorems 2 and 3 are also fuzzy based models of system (1). But, Theorem 1 is the most important case. Because, the zero-covid policy dependents on the number of new cases.

Mathematically, we try to prove a case that the existence of even single case in city **A** can lead to the chaotic attitude not only in city **A** but in the nearby cities **B**, **C** etc as well.

## Possibility of disease spreading during zero-covid policy

We have used the concept of multi-scroll attractors in this section to prove and visualize the possible chances of covid spreading between two cities. However, the two cities **A** and **B** are considered in a sense that city **A** has some cases of covid, while city **B** is free from covid. For this purpose, we used the saw−tooth pulse signal function in system (1) following the steps of Algorithm 1.

### Generation of multi-scroll attractors due to the number of new cases

We designed a scheme with the aid of Algorithm 1 and saw-tooth pulse signal (24) in which the number of attractors start increasing rapidly not only in the current city but in the nearby cities as well. In this case, the state variable $x_{1}$ is in the command of a pulse signal function. Systems (7) and (8) can be rewritten as:

13 $$\begin{aligned} \begin{pmatrix} \dot{x}_{1} \\ \dot{x}_{2} \\ \dot{x}_{3} \end{pmatrix}= \begin{pmatrix} -\alpha _{4} &{} \alpha _{6} M_{i} &{} \alpha _{1}+\alpha _{5} M_{i}\\ 0 &{} -\beta _{2} M_{i} &{} 0\\ 0 &{} -\gamma _{2}M_{i} &{} \gamma _{1}-\gamma _{3}M_{i} \end{pmatrix} \begin{pmatrix} \sigma _{x1} \\ {x}_{2} \\ {x}_{3} \end{pmatrix} + \begin{pmatrix} -\alpha _{2}x_{3}^2 + \alpha _{3}x_{2}x_{3}\\ \beta _{1} x_{2} x_{3} \\ \gamma _{4} \sigma _{x1}^2 \end{pmatrix} \end{aligned}$$

for $i=1,2$, where $\sigma _{x1}$=$x_{1}-\omega _{1} f(t)$ such that $\omega _{1} \in \mathbb {R}$ and can generate multiple scrolls in $x_{1}$ direction by setting the control parameter values $\zeta _{x}=0.25$, $\mho _{z}=0.5$, $K_{z}=7$ accordingly. Setting Eq. (1) equals to zero, we get the following two equilibrium points;

14 $$\begin{aligned} {\left\{ \begin{array}{ll} E_{0}=(0,\hspace{5.0pt}0,\hspace{5.0pt}0),\\ E_{1}=(\frac{\beta _{1}E_{1c}}{\beta _{2}},-\hspace{5.0pt}E_{1b},\hspace{5.0pt}E_{1c}), \end{array}\right. } \end{aligned}$$

where $E_{1b}=\left( \frac{m_{11}m_{23}-m_{13}m_{21}}{m_{12}m_{23}-m_{13}m_{22}}\right)$ and $E_{1c}=\left( \frac{m_{11}m_{22}-m_{12}m_{21}}{m_{12}m_{23}-m_{13}m_{22}}\right)$ with

15 $$\begin{aligned} {\left\{ \begin{array}{ll} m_{11}=\alpha _{1}-\frac{\alpha _{4}\beta _{1}}{\beta _{2}}, \hspace{5.0pt}m_{21}=\alpha _{1}{,}\\ m_{12}=\alpha _{3}-\frac{\alpha _{6}\beta _{1}}{\beta _{2}}, \hspace{5.0pt}m_{22}=-\left( \gamma _{2}+\frac{\gamma _{3}\beta _{1}}{\beta _{2}}\right) {,}\\ m_{13}=-\alpha _{2}+\frac{\alpha _{5}\beta _{1}}{\beta _{2}}, \hspace{5.0pt}m_{23}=\gamma _{4}\left( \frac{\beta _{1}^2}{\beta _{2}^2}\right) . \end{array}\right. } \end{aligned}$$

**Figure 2:**
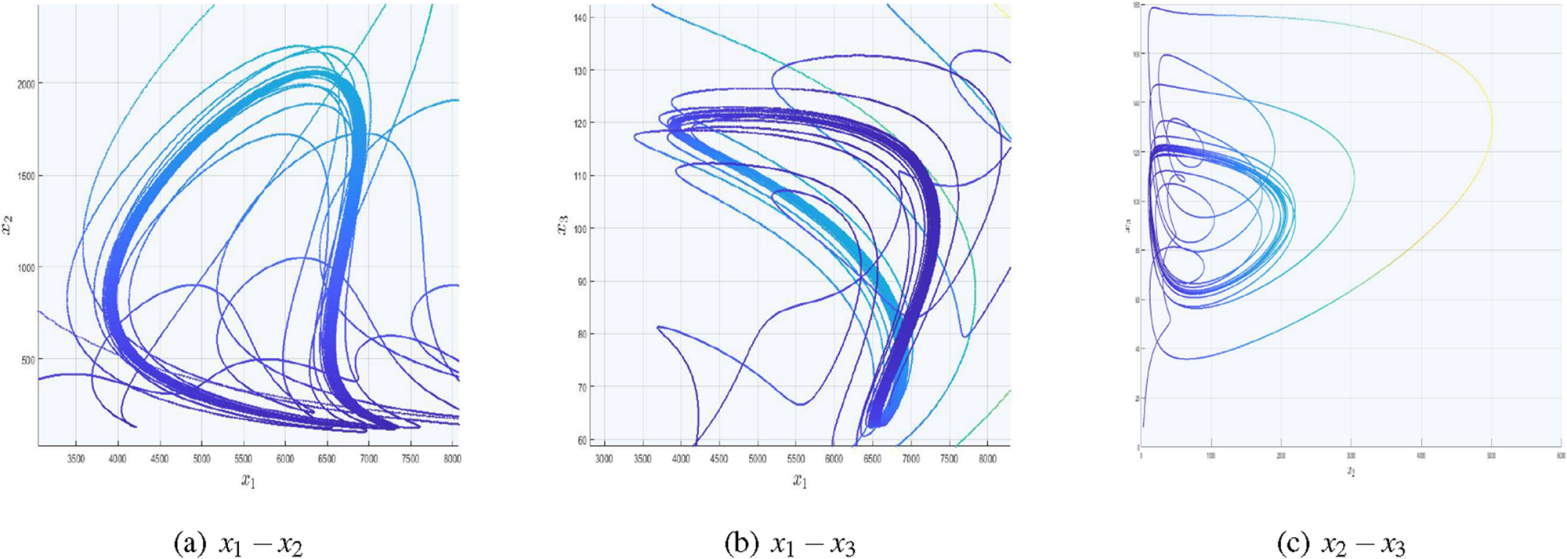
Impact of $x_{1}$ with the inclusion of signal pulsed function in $x_{1}-$direction.

**Figure 3:**
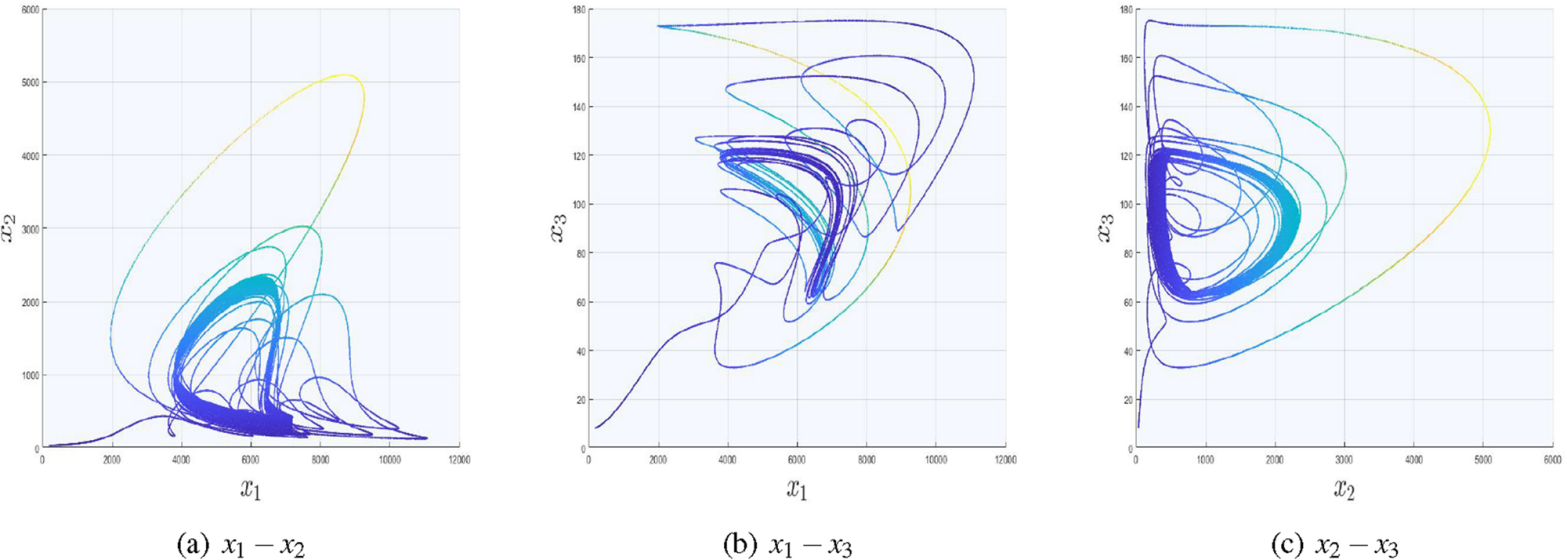
Impact of $x_{2}$ with the inclusion of signal pulsed function in $x_{2}-$direction.

**Figure 4:**
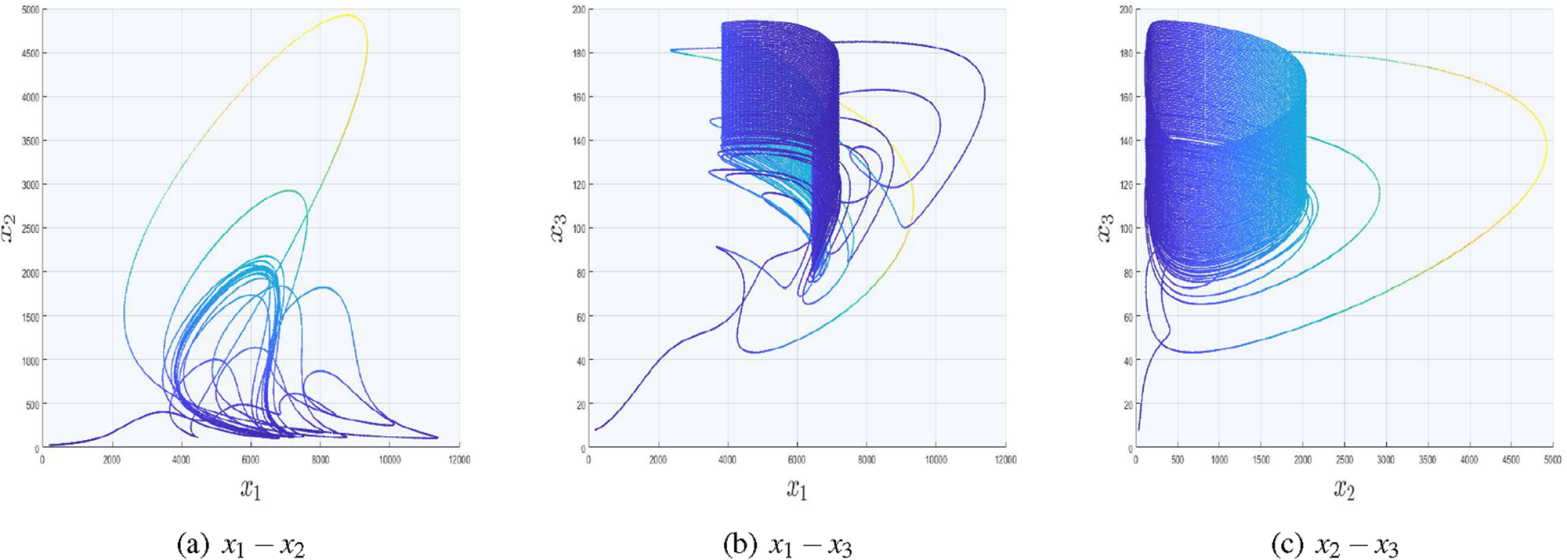
Impact of $x_{3}$ with the inclusion of signal pulsed function in $x_{3}-$direction.

$E_{0}$ is the genuine case and will be achieved with the vanishing of covid permanently from all over the world, while $E_{1}$ is the unique non−zero equilibria of system (1). Substituting signal function (23) into system (1) in *x* direction

16 $$\begin{aligned}&E_{1}=\left( \left( \frac{\beta _{1}}{\beta _{2}}\right) n_{x} E_{1c}, -E_{1b}, E_{1c}\right) ,\\&{n_{x}=\{0,\hspace{5.0pt}\pm 1,\hspace{5.0pt}\pm 2,\hspace{5.0pt}\pm 3,\hspace{5.0pt}\pm 4,\hspace{5.0pt}\pm 5,\hspace{5.0pt}\pm 6,\hspace{5.0pt}\pm 7\}}\nonumber \end{aligned}$$

leads to the generation of 15 equilibrium points for $K_{x}=7$, which is also visible in Fig. 2. Additionally, this picture is comprised of three sub-figures in which the signal pulse function is injected by substituting the first variable. It can be seen that the paths in the $x_1-x_2$ and $x_1-x_3$ planes got more dense. This feature indicates the scroll’s emergence in the $x_{1}$ direction. However, as seen in Fig. 2c, the phase portrait is comparable to the original COVID-19 system. Similarly, the covid models with $x_{2}$ and $x_{3}$ substitutions are presented in Table 3, while its phase portraits are depicted in Figs. (3 and 4), respectively.

**Table 3:** Covid system with multi-scroll attractors in $x_{1}$ direction.

System equations	$\dot{x}_{1}=-\alpha _{4} x_{1}+ \alpha _{6} M_{i} x_{1}+$	$\dot{x}_{1}=-\alpha _{4} {x_{1}}+ \alpha _{6} M_{i} {x_{1}}+$
	$(\alpha _{1}+\alpha _{5} M_{i})x_{1}-\alpha _{2}x_{3}^2 + \alpha _{3} \sigma _{x2}x_{3},$	$(\alpha _{1}+\alpha _{5} M_{i}){x_{1}}-\alpha _{2}\sigma _{x3}^2 + \alpha _{3}x_{2}\sigma _{x3},$
	$\dot{x}_{2}=-\beta _{2} M_{i} \sigma _{x2}+\beta _{1} \sigma _{x2} x_{3},$	$\dot{x}_{2}=-\beta _{2} M_{i} x_{2}+\beta _{1} x_{2} \sigma _{x3},$
	$\dot{x}_{3} = -\gamma _{2}M_{i} x_{3} + (\gamma _{1}-\gamma _{3}M_{i})x_{3} + \gamma _{4} x_{1}^2,$	$\dot{x}_{3} = -\gamma _{2}M_{i} \sigma _{x3} + (\gamma _{1}-\gamma _{3}M_{i})\sigma _{x3}+\gamma _{4} x_{1}^2$,
Control parameters	$\zeta _{y}=0.125$,$\mho _{y}=0.7$, $K_{y}=3$	$\zeta _{z}=0.85$, $\mho _{z}=0.15$, $K_{z}=2$

As in Fig. 3, $x_{2}$ is substituted with the signal function; hence, the influence of $x_2$ on the other two state variables and the appearance of scrolls are depicted in Fig. 3a,c. However, Fig. 3b is independent on $x_{2}$, hence its trajectories are devoid of scrolls.

Figure 4 shows influence of the replacement of the third state variable with the considered signal function on the other two state variables.

### Generation of multi-scroll attractors due to the number of new cases and severe cases

In previous case, we have fixed one variable $x_{1}$ and replaced it with pulse projection signal control input $\sigma _{t}$. In this sub-portion, we fix two states $\{x_{1},x_{2}\}$ and replace them with their corresponding signal pulse control inputs $\{\sigma _{x1},\sigma _{x2}\}$. Due to these changes systems (7) and (8) can adobe the form:

17 $$\begin{aligned} \begin{pmatrix} \dot{x}_{1} \\ \dot{x}_{2} \\ \dot{x}_{3} \end{pmatrix}= \begin{pmatrix} -\alpha _{4} &{} \alpha _{6} M_{i} &{} \alpha _{1}+\alpha _{5} M_{i}\\ 0 &{} -\beta _{2} M_{i} &{} 0\\ 0 &{} -\gamma _{2}M_{i} &{} \gamma _{1}-\gamma _{3}M_{i} \end{pmatrix} \begin{pmatrix} \sigma _{x1} \\ \sigma _{x2} \\ {x}_{3} \end{pmatrix} + \begin{pmatrix} -\alpha _{2}x_{3}^2 + \alpha _{3}\sigma _{x2}x_{3}\\ \beta _{1} x_{3} \sigma _{x2} \\ \gamma _{4} \sigma _{x1}^2 \end{pmatrix} \end{aligned}$$

for $i=1,2$, where $\sigma _{x1}$ = $x_{1}-\omega _{1} f(t)$, $\sigma _{x2}$ = $x_{2}-\omega _{2} f(t)$ and can generate multiple scrolls in $x_{1}-x_{2}$ plane.

18 $$\begin{aligned}&E_{1}=\left( \left( \frac{\beta _{1}}{\beta _{2}}\right) n_{x} E_{1c}, -n_{y}E_{1b} , E_{1c}\right) ,\nonumber \\&{n_{x}=\{0,\hspace{5.0pt}\pm 1,\hspace{5.0pt}\pm 2,\hspace{5.0pt}\pm 3,\hspace{5.0pt}\pm 4,\hspace{5.0pt}\pm 5,\hspace{5.0pt}\pm 6,\hspace{5.0pt}\pm 7\},}\nonumber \\&{n_{y}=\{0,\hspace{5.0pt}\pm 1,\hspace{5.0pt}\pm 2,\hspace{5.0pt}\pm 3,\hspace{5.0pt}\pm 4\}.} \end{aligned}$$

**Figure 5:**
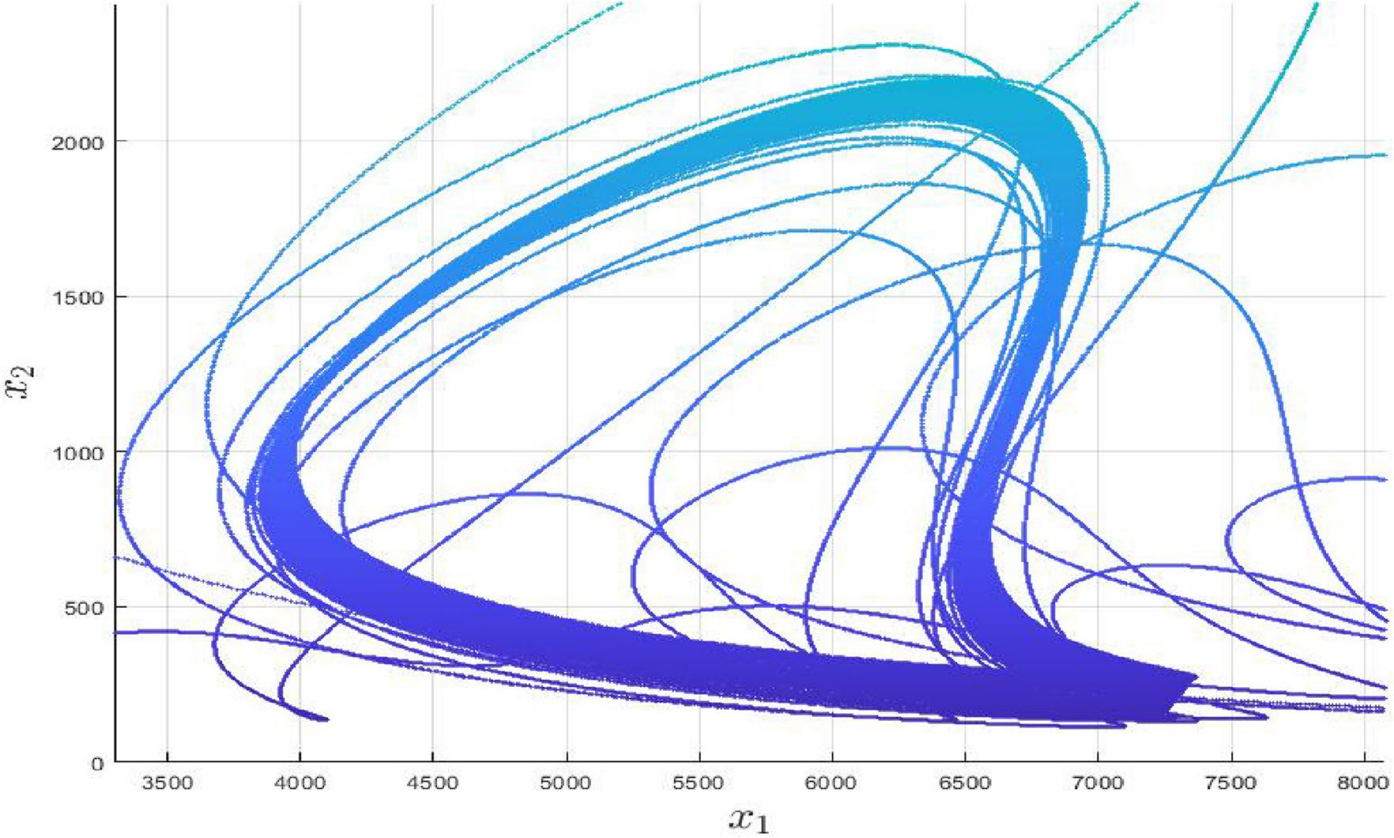
The occurrence of chaos in fuzzy based covid−19 system.

In Fig. 5, we have included two saw−tooth functions with the replacement of $x_{1}$ and $x_{2}$, while the third variable remains unchanged. Moreover, we have used $(\zeta _{x},\mho _{x},K_{x})=(0.25,0.5,7)$ and $(\zeta _{y},\mho _{y},K_{y})=(0.125,0.7,3)$ as control parameters using signal function (23). Due to the inclusion of signal functions in *x* and *y* directions, one can get $(15\times 9 )$ number of equilibrium points. The rest of two cases for the generation of multi-scrolls in $x_{1}-x_{3}$ and $x_{2}-x_{3}$ planes along with their phase portraits and control parameters are given in Table 4. It is important to mention here that, the graph in Fig. 3a looks similar as Fig. 5 but in fact the difference is trajectories in Fig. 5 are slightly tilted towards $x_{1}$ direction due to the inclusion of signal function in that direction as well.

**Table 4:** Two dimensional multi-scrolls in system (1).

System equations	$\dot{x}_{1}=-\alpha _{4} \sigma _{x1}+ \alpha _{6} M_{i} \sigma _{x1}+$	$\dot{x}_{1}=-\alpha _{4} {x_{1}}+ \alpha _{6} M_{i} {x_{1}}+$
	$(\alpha _{1}+\alpha _{5} M_{i})\sigma _{x1}-\alpha _{2}\sigma _{x3}^2 + \alpha _{3} x_{2}\sigma _{x3},$	$(\alpha _{1}+\alpha _{5} M_{i}){x_{1}}-\alpha _{2}\sigma _{x3}^2 + \alpha _{3}\sigma _{x2}\sigma _{x3},$
	$\dot{x}_{2}=-\beta _{2} M_{i} {x_{2}}+\beta _{1} x_{2} \sigma _{x3},$	$\dot{x}_{2}=-\beta _{2} M_{i} \sigma _{x2}+\beta _{1} \sigma _{x2} \sigma _{x3},$
	$\dot{x}_{3} = -\gamma _{2}M_{i} \sigma _{x3} + (\gamma _{1}-\gamma _{3}M_{i})\sigma _{x3} + \gamma _{4} \sigma _{x1}^2$,	$\dot{x}_{3} = -\gamma _{2}M_{i} \sigma _{x3} + (\gamma _{1}-\gamma _{3}M_{i})\sigma _{x3} + \gamma _{4} x_{1}^2,$
Control parameters	$\zeta _{x}=0.25$,$\mho _{x}=0.5$,$K_{x}=7.$	$\zeta _{y}=0.125$,$\mho _{y}=0.7$,$K_{y}=3.$
	$\zeta _{z}=0.85$,$\mho _{z}=0.15$,$K_{z}=2.$	$\zeta _{z}=0.85$,$\mho _{z}=0.15$,$K_{z}=2.$
Phase portraits		

### Generation of three dimensional multi-scroll attractors

**Figure 6:**
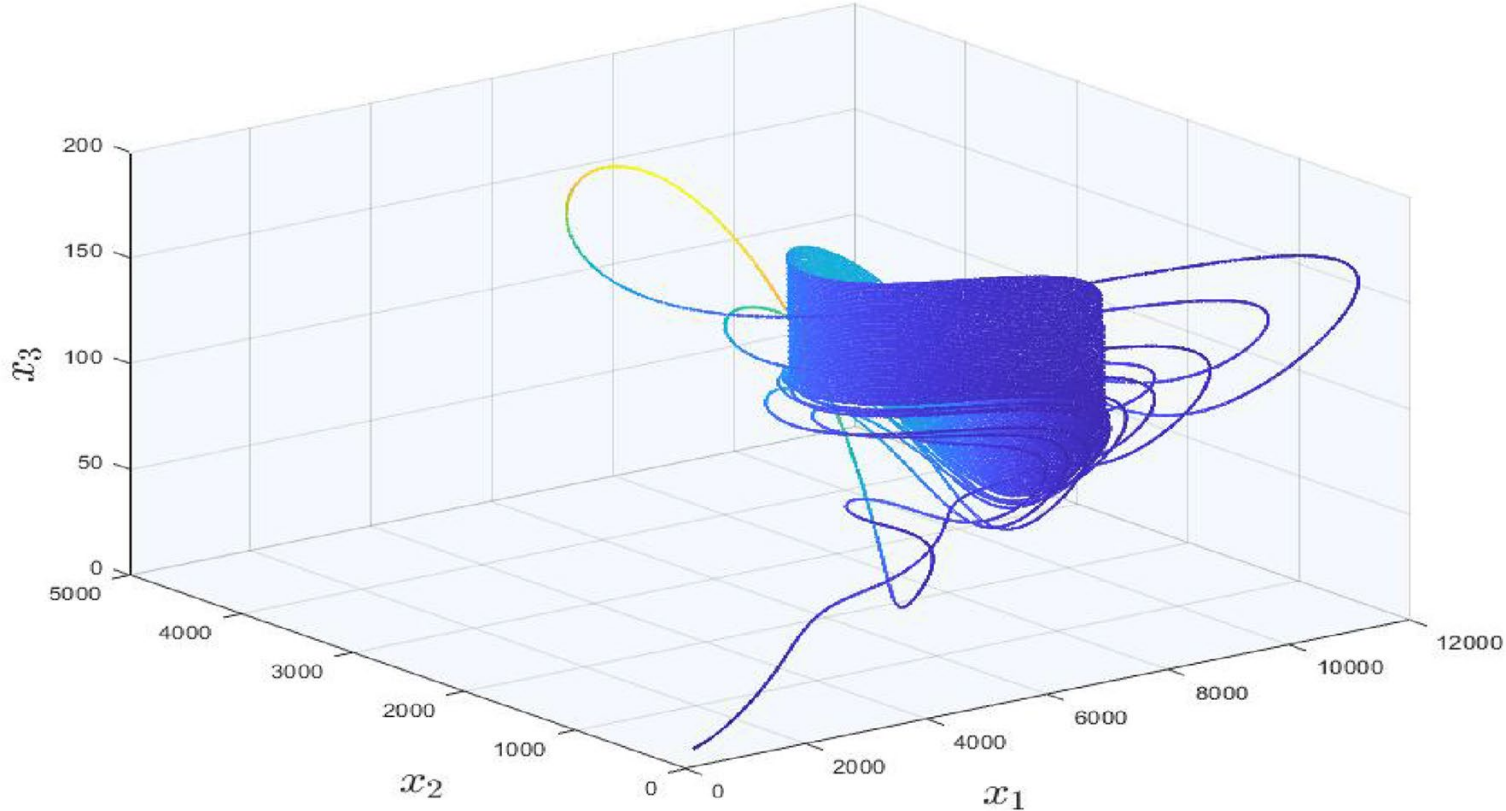
Three dimensional multi-scroll attractors in covid-19 system.

The complete picture of three dimensional multi-scroll attractors in our considered model can be seen in Fig. 6. To achieve this case, one can need to follow the steps of Algorithm 1 for three directions. The considered system can be transformed into the following form:

19 $$\begin{aligned}&\dot{x}_{1}=(\alpha _{1}+\alpha _{5} M_{i})\sigma _{x1}-\alpha _{2}\sigma _{x3}^2 + \alpha _{3} \sigma _{x2}\sigma _{x3}-\alpha _{4} \sigma _{x1}+ \alpha _{6} M_{i} \sigma _{x1},\nonumber \\&\dot{x}_{2}=\beta _{1} \sigma _{x2} \sigma _{x3}-\beta _{2} M_{i} {\sigma _{x2}},\nonumber \\&\dot{x}_{3} = (\gamma _{1}-\gamma _{2}M_{i}-\gamma _{3}M_{i})\sigma _{x3} + \gamma _{4} \sigma _{x1}^2, \end{aligned}$$

where $\sigma _{xi}$ $=$ $x_{i}-\omega _{i}f_{i}(t)$; $i=1,2,3$. In our case, we have selected $\omega _{1}=\omega _{2}=\omega _{3}=1$ but there is no restriction on the value of $\omega _{i};\hspace{5.0pt}i=1,2,3$. If the values of $\mathbf {\omega }$ are selected more than $\textbf{1}$, then the trajectories will be more denser and for $\mathbf {\omega }=\textbf{0}$ there will be no scroll attractors.

20 $$\begin{aligned}&E_{1}=\left( \left( \frac{\beta _{1}}{\beta _{2}}\right) n_{x} E_{1c}, - n_{y}E_{1b} , n_{z}E_{1c}\right) ,\nonumber \\&{n_{x}=\{0,\hspace{5.0pt}\pm 1,\hspace{5.0pt}\pm 2,\hspace{5.0pt}\pm 3,\hspace{5.0pt}\pm 4,\hspace{5.0pt}\pm 5,\hspace{5.0pt}\pm 6,\hspace{5.0pt}\pm 7\},}\nonumber \\&{n_{y}=\{0,\hspace{5.0pt}\pm 1,\hspace{5.0pt}\pm 2,\hspace{5.0pt}\pm 3,\hspace{5.0pt}\pm 4\},}\nonumber \\&{n_{z}=\{0,\hspace{5.0pt}\pm 1,\hspace{5.0pt}\pm 2\}}. \end{aligned}$$

$E_{1b}$ and $E_{1c}$ are defined in Eqs. (14 and 15), whereas Eq. (20) indicates the injection of three signal functions in *x*, *y* and *z* directions respectively and can produce $(15\times 9 \times 5)$ number of equilibrium points.

## Discussion

In the preceding section, we proved and explained that trajectories of our considered model are still moving towards weird attractor even if the number of new cases each day is decreased to the interval (0, 1) with the aid of fuzzy theory, whereas we used the saw-tooth function to prove the existence of multi-scrolls. By combining the information in these two parts, we were able to demonstrate that corona is an eternal phenomenon. For ease, we have explained the concept between two nearby cities. Globally, if the same concept is adopted, then the results will be same for the whole world as well.

**Figure 7:**
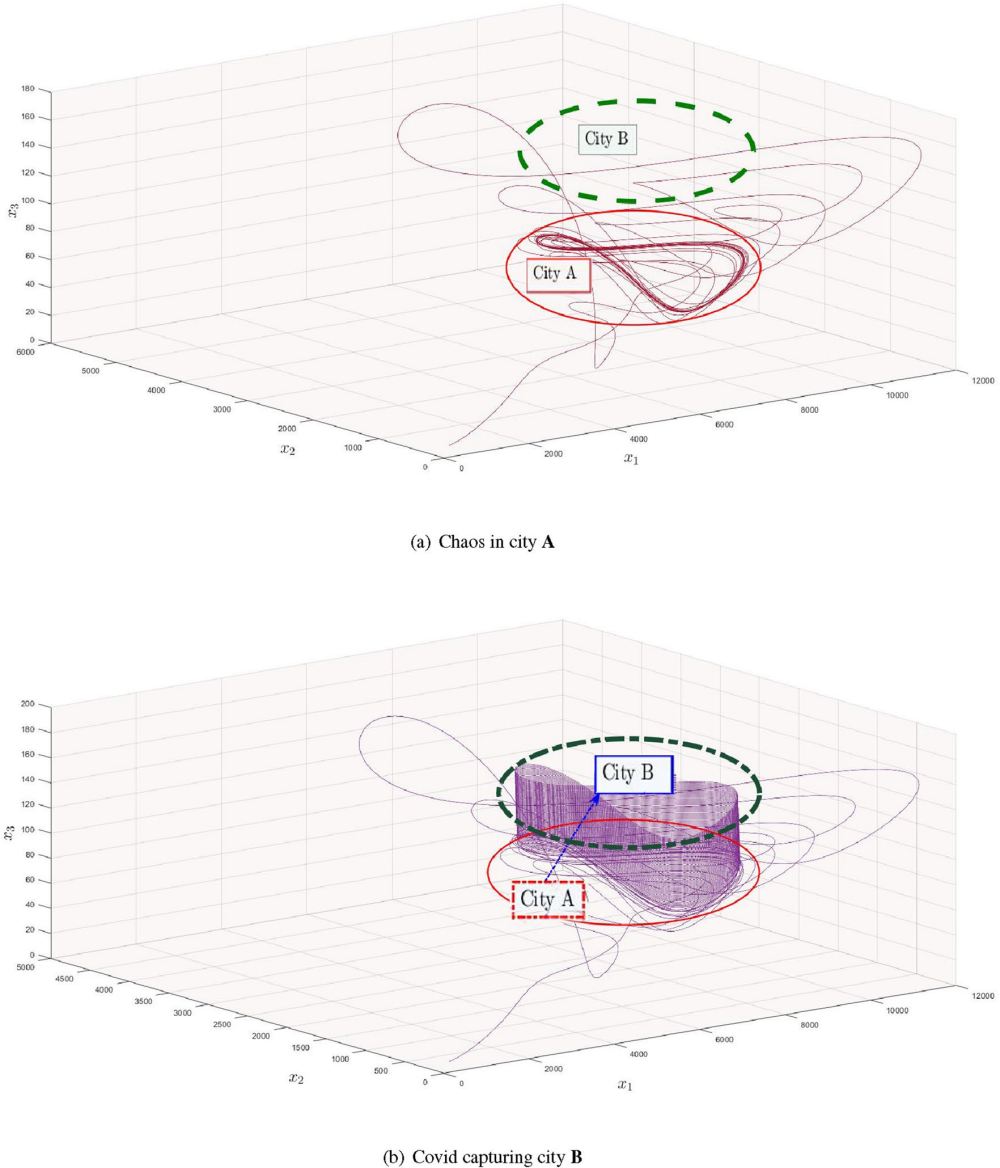
Appearance of covid in neighboring city due to intercity transportation.

In Fig. 7a, we explore a situation with two cities, **A** and **B**, to better illustrate our point. City **A** is encircled with red lines and is supposed to be a location, where there is corona and have unpredictable situation, whereas city **B** is encircled with green doted lines and is considered as a place where there are zero cases and ensure us that the situation is normal. Jumping to Fig. 7b, one can observe a blue line heading from city **A** towards **B**. After some time, the situation in city **B** becomes alarming and unpredictable trajectories emerge there. However, city **A** and **B** both are taken as arbitrary and one can also consider the link between two countries, one with zero cases and other with the existence of corona cases. If the routes of such type of countries are open for each other, then according to our obtained results, the pulses will emerge somewhere inside the country with zero cases and again the situation will be alarming. The following points are declared as the remedy to achieve zero cases per day between two cities or countries.

- City/ Country **B** with zero cases should close all their borders for travel and trade purpose from the rest of covid active cases countries.
- City/ Country **B** should also ban on their inter-provincial trading and traveling.
- City/ Country **B** should follow the complete lock down in all over the country at a time for at least 14 days.

But, there are following consequences

- City/ Country **B** can suffer a lot of loss in business and inter-bank marketing.
- The peoples inside city/ country **B** can suffer anxiety, mental problems and unemployment, while the investors in city/ country **B** can stop their business and can move to other places.
- City/ Country **B** can face droughtiness and deprive of resources.

which are not only dangerous for their own people but for the rest of world as well. In March, 2022, New zealand achieved the zero-covid in rest of the country for only three days. But, due to the bad impact and consequences of strict restrictions they got many loss. In long term, China was following the zero-covid policy and had controlled this pandemic disease, according to their population, in systematic way but still the situation in some provinces of China was alarming and leading to the complete lock-down.

**Figure 8:**
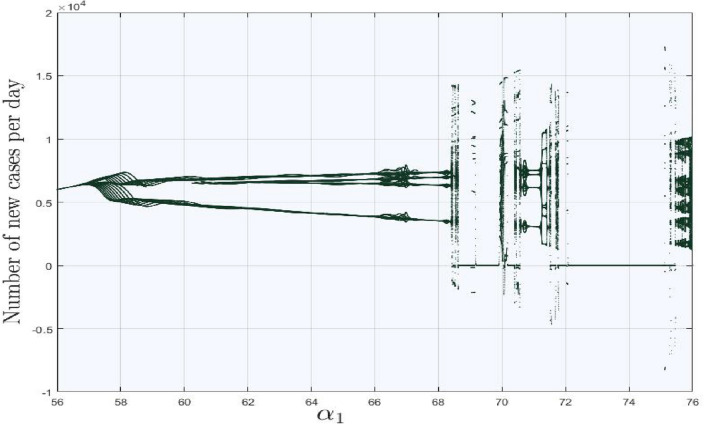
Generation of number of new cases per day with respect to bifurcation parameter $\alpha _{1}$ in city **B**.

**Figure 9:**
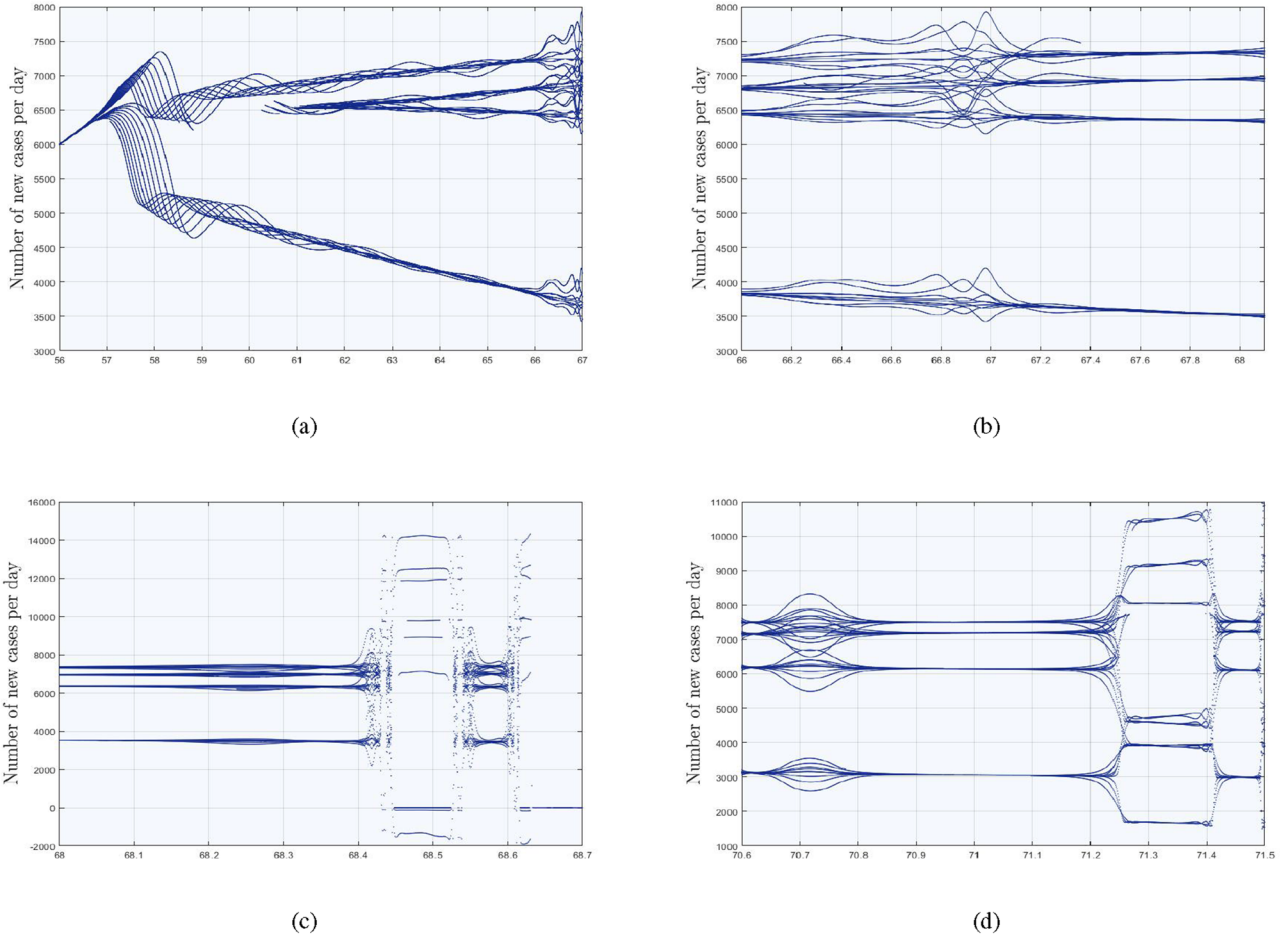
Creation of alarming situation in city **B**.

The generic number of new cases per day in city **B** is plotted with respect to $\alpha _{1}$ ranging from 56 to 76. In Fig. 8 it can be seen more clearly that as $\alpha _{1}$ reaches to 57, bifurcation which approaches to unpredictable behavior by moving $\alpha _{1}$ to right side. However, in between of Fig. 8, some fluctuations are observed but at the end the system still remain chaotic. Figures 9($a-d$) are the sub-portions of Fig. 8 and are zoomed for further explanation in depth. Starting from Fig. 9(*a*), twenty trajectories emerges at a time from a single point at $\alpha _{1}=57$ and adopt the chaotic attitude for $\alpha _{1}=66$, can be seen in Fig. 9(*b*). Moreover, Fig. 9(*c*) is the sub-portion, where the sudden change in the number of new cases are observed, but as we move forward, it shows us the chaotic attitude once again. These figures explain that covid will remain till end and can never vanishes like simple influenza. Although, due to usage of vaccinations, booster and its medicines, an infected person can be cured but remains somewhere in his body and emerges with respect to suitable environment.

## Methods

This section comprise of methodologies and our designed algorithm, that are used for the better understanding of this work.

### Definition 1

(^34^) A membership function $\Gamma _{G}$ of fuzzy set *G* is a mapping:

$$\begin{aligned} \Gamma _{G}: \hspace{5.0pt}\Omega \rightarrow [0,1],\hspace{5.0pt}\forall z\in \Omega \end{aligned}$$

has a membership degree $\Gamma _{G}(z)\in [0,1]$. In other-words, *G* can be determined as $G=\{z \in \Omega / (z,\Gamma _{G}(z))\}$.

### Definition 2

(^34^) Let us suppose the $j^{th}$ weight $u_{j}$ and $\Gamma _{G}(z)$ the membership degree of fuzzy set *G*. Then, the weighted sum is defined as:

$$\begin{aligned} h(z)=\frac{\sum _{j=1}^{N} u_{j} h_{j}}{\sum _{j=1}^{N} u_{j}}. \end{aligned}$$

### Lemma 1

(^34^) *Let us suppose there exist the following k-nonlinear term:*

21 $$\begin{aligned} h_{k}=z_{1} z_{2} \cdots z_{k} \end{aligned}$$

in any dynamical system. Then, the nonlinear term (21) can be represented as a linear weighted sum of the form

22 $$\begin{aligned} h_k=\left( \sum _{j_2, j_3, \ldots , j_k=1}^2 \upsilon _{j_2 j_3 \cdots j_k} \cdot \kappa _{j_2 j_3 \cdots j_n}\right) z_1, \end{aligned}$$

where

$$\begin{aligned} \kappa _{j_2 j_3 \cdots j_k}=\prod _{p=2}^k M_{j_p}^p, \quad \upsilon _{j_2 j_3 \cdots j_k}=\prod _{p=2}^k \Gamma _{j_p}^p. \end{aligned}$$

$\Gamma _{j_p}^p$ in Eq. (22) is positive semi−definite for all *z*.

A pulse signal based function of the form:

23 $$\begin{aligned} h_1(z)=\zeta \left\{ z-\mho _1\left[ -{\text {sgn}}(z)+\sum _{i=0}^{K-1}\left( {\text {sgn}}\left( z+2 i \mho _1\right) +{\text {sgn}}\left( z-2 i \mho _1\right) \right) \right] \right\} \end{aligned}$$

or

24 $$\begin{aligned} h_2(z)=\zeta \left\{ z-\mho _1\left[ \sum _{i=0}^{K-1}\left( {\text {sgn}}\left( z+(2 i+1) \mho _1\right) +{\text {sgn}}\left( z-(2 i+1) \mho _1\right) \right) \right] \right\} \end{aligned}$$

is termed as a saw-tooth function^45^ in engineering field with the constants $\zeta >0$, $K\ge 1$, $\mho _{1}>0$ and

25 $$\begin{aligned} {\text {sgn}}(z)={\left\{ \begin{array}{ll} -1: \quad \quad z<1,\\ \hspace{5.0pt}0: \quad \quad \hspace{5.0pt}z=0,\\ +1: \quad \quad z>1. \end{array}\right. } \end{aligned}$$

**Figure 10:**
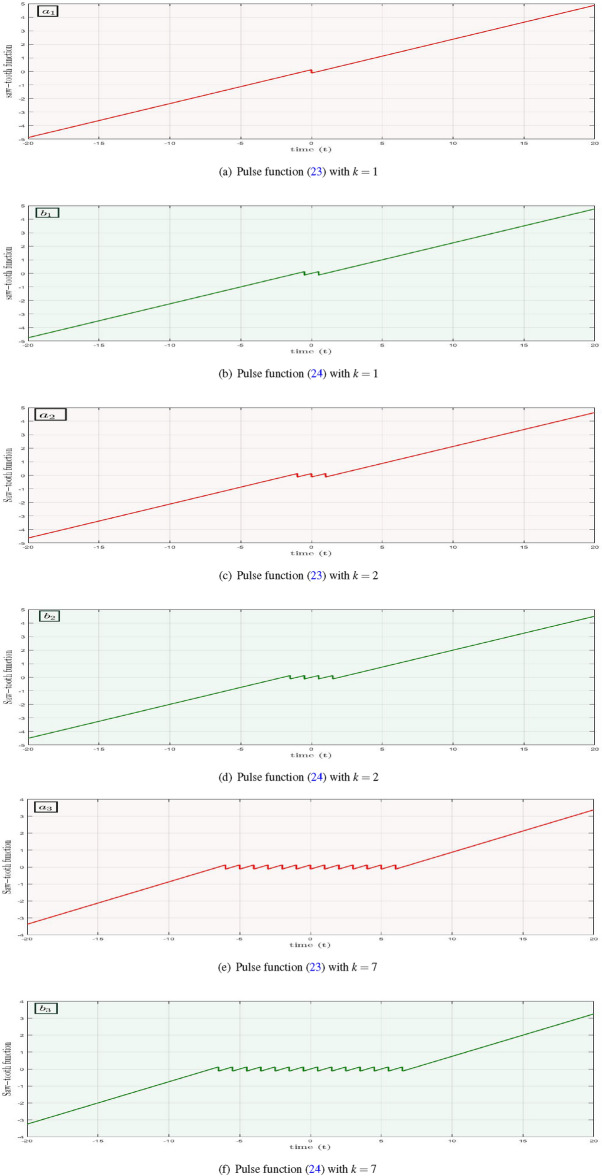
Generation of pulses for different values of *k* with $\zeta =0.25$ and $\mho _{1}=0.5$.

However, both the signal functions are same but Eq. (23) is used for the generation of 2*n* pulses, while Eq. (24) is used for $2n+1$ pulses. But throughout this paper, we have used Eq. (23) as a signal function for the generation of multi-scrolls in considered model. Figure 10 consists of scrolls using equation (23) on left side in red color, whereas the green colored pulses on right side are scrolls using equation (24). Moreover, Eq. (25) is the signum function and has an important role in the creation of various signal functions in engineering. For more clarification, we select random values of *k* for the generation of saw−tooth signals in the time range of $t=[-20,20]$.

Readers can follow Algorithm 1 to generate multi-scrolls not only in the model considered here but can also be implemented in all type of systems to achieve desired results. $\omega _{i};\,i=1,2,3$ are included in the procedure for the first time and is nowhere used in the literature for multi-scroll attractors. These parameters help as a tuner button in the Algorithm 1.

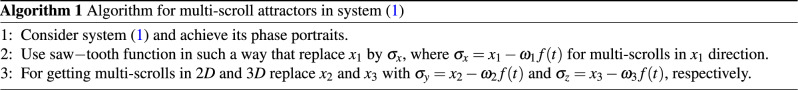



### Remark 2

Let us consider $\omega _{1}$ = $\omega _{2}$  = $\omega _{3}$  = 0 in Algorithm 1 then, system (1) does not contain any scrolls.

### Remark 3

Suppose $\omega _{2}$ = $\omega _{3}$ = 0 in Algorithm 1 then, system (1) contain scrolls about $x_{1}-$axis.

### Remark 4

Using $\omega _{3}$ = 0 in Algorithm 1 leads system (1) to the existence of scrolls about $x_{1}$ and $x_{2}$ axes.

### Remark 5

There exist multi-scroll attractors in three dimensions for all $\omega _{i}\ne 0$.

Signals plotted in Fig. 10 for different values of *k* have significant role in the field of dynamical systems. These signals can be embedded in system of differential equations to help in the generation of multi-scrolls. Moreover, the number of equilibrium points in any dynamical system has a directly proportional relation with the number of *k* in signal functions (23) or (24).

## Data Availability

The datasets used and/or analyzed during the current study available from the corresponding author on reasonable request.
